# Replication Stress and Consequential Instability of the Genome and Epigenome

**DOI:** 10.3390/molecules24213870

**Published:** 2019-10-27

**Authors:** Pawlos S. Tsegay, Yanhao Lai, Yuan Liu

**Affiliations:** 1 Biochemistry Ph.D. Program, Florida International University, Miami, FL 33199, USA; ptseg001@fiu.edu; 2 Department of Chemistry and Biochemistry, Florida International University, 11200 SW 8th Street, Miami, FL 33199, USA; yalai@fiu.edu; 3 Biomolecular Sciences Institute, Florida International University, Miami, FL 33199, USA

**Keywords:** oxidative DNA damage, DNA replication stress, replication fork stalling, genomic and epigenomic instability, DNA methylation, histone modifications, miRNAs

## Abstract

Cells must faithfully duplicate their DNA in the genome to pass their genetic information to the daughter cells. To maintain genomic stability and integrity, double-strand DNA has to be replicated in a strictly regulated manner, ensuring the accuracy of its copy number, integrity and epigenetic modifications. However, DNA is constantly under the attack of DNA damage, among which oxidative DNA damage is the one that most frequently occurs, and can alter the accuracy of DNA replication, integrity and epigenetic features, resulting in DNA replication stress and subsequent genome and epigenome instability. In this review, we summarize DNA damage-induced replication stress, the formation of DNA secondary structures, peculiar epigenetic modifications and cellular responses to the stress and their impact on the instability of the genome and epigenome mainly in eukaryotic cells.

## 1. Introduction

**Figure 1:**
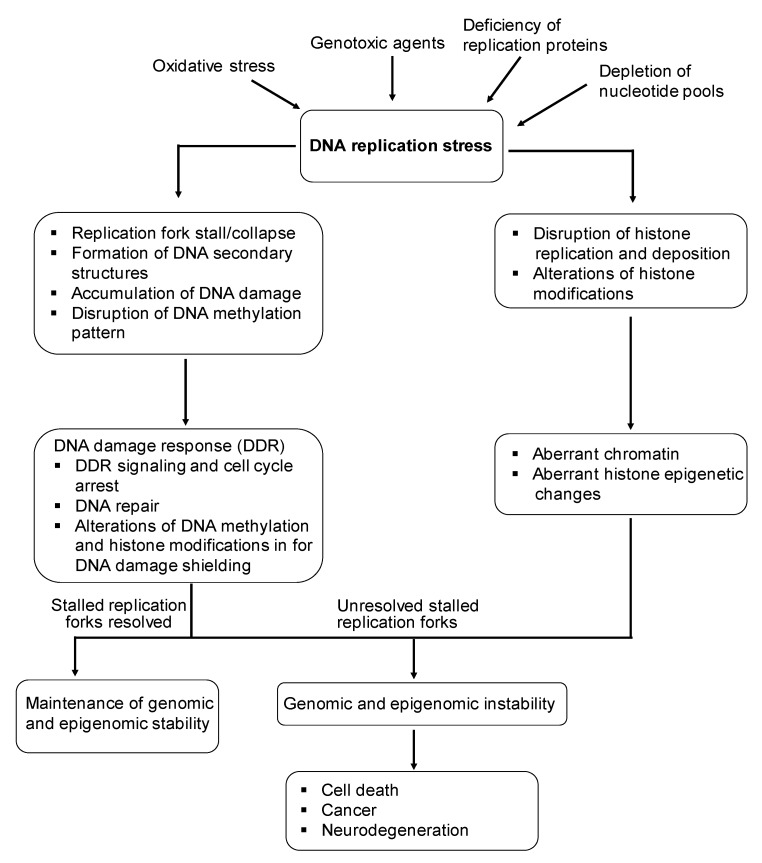
DNA replication stress leads to genomic and epigenomic instability associated with diseases.

Faithful copying of genetic information is vital for cells to maintain genomic and epigenomic stability. During cell division, DNA replication includes the replication of DNA, the DNA methylation pattern, as well as the duplication of histones and their modifications. These allow the genetic and epigenetic information of a cell to be copied and passed to daughter cells. Also, the integrity of the genome and epigenome in cells is maintained through the coordination between DNA replication and cell cycle, which contains the G1, S, G2 and M phases, respectively [1,2,3]. Replication of the entire genome and its epigenetic modifications, along with the replication of histones and their modifications have to be completed during the S phase before the cell cycle can enter its M phase, where one single cell is divided into two daughter cells. However, during DNA replication, the opened genomic DNA is also susceptible to attack by varieties of DNA damage. This can lead to replication stress, i.e., replication fork stalling that subsequently results in the accumulation of DNA damage and the formation of secondary DNA structures, triggering DNA damage response (DDR) and repair, as well as corresponding epigenetic changes. All these processes can alter the effectiveness and precision of DNA replication, causing genome and epigenome instability that can ultimately lead to human diseases such as cancer [4,5] (Figure 1).

## 2. DNA Replication

The bidirectional DNA replication in eukaryotes starts at multiple replication initiation sites, known as replication origin, that encompasses the DNA sequences recognized and bound by the replication initiator proteins [6]. Subsequently, DNA helicase complex is formed at the replication origin through the assembly of the head to head double hexamer minichromosome maintenance protein (MCM) with the help of cell division cycle 6 (Cdc6), Cdc10-dependent transcript 1 (Cdt1), and origin recognition complex (ORC) [7,8,9] in the G1 phase of the cell cycle [7,9,10]. The double hexamer MCM helicase complex is then activated in the S phase by cyclin-dependent kinase (CDK) and Dbf4 dependent kinase (DDK) [11,12,13,14,15], forming the functional helicase complex, cell division cycle 45 (CDC45)-MCM-GINS, (CMG complex helicase) with CDC45, and GINS [11,16,17,18]. Double helical DNA is then unwound upon the recruitment of CMG complex helicase, resulting in the formation of replication forks [12,19,20,21]. In this process, the ATP-dependent MCM complex serves as the motor of DNA replication by unwinding double-strand DNA. 

Unwound single-stranded DNA (ssDNA) is then bound by the ssDNA binding protein, replication protein A (RPA) for protection from the degradation and formation of secondary structures [22,23]. Then the pol α-primase complex is recruited to the replication forks through its interaction with the chromosome transmission fidelity 4 (Ctf4) protein that also interacts with the GINS in the CMG complex [24]. Since DNA is synthesized in a 5’ to 3’ direction during replication, the DNA synthesis that is carried out by DNA polymerase ε (pol ε) in the leading strand is continuous, whereas the DNA synthesis by DNA polymerase δ (pol δ) on the lagging strand is discontinuous with the synthesis of short Okazaki fragments with 100–250 nucleotides. Both pol ε and pol δ interact with the replication cofactor, proliferating cell nuclear antigen (PCNA), which is loaded on the double-strand DNA by the clamp loader protein complex, replication factor C (RFC). PCNA anchors the polymerases to the template strand, allowing the polymerases to perform the processive DNA synthesis [25,26] and ensuring the high efficiency of DNA replication. Finally, RNA primers in the Okazaki fragments are removed by RNase HI [27] and flap endonuclease 1 (FEN1) [28,29]. Replicative DNA polymerase pol δ also plays a role in removing RNA primers in the Okazaki fragments by coordinating with FEN1 flap cleavage in that pol δ strand displacement synthesis creates a flap containing an RNA primer, which is then cleaved by FEN1 flap cleavage [30,31]. The generated nicked DNA is then sealed by DNA ligase I [32], thereby leading to the completion of DNA replication. While replicative DNA polymerases exhibit a high efficiency of DNA synthesis, they also have a high fidelity of incorporating correct nucleotides. This is because these polymerases bear a catalytic site with a rigid structure, and have their 3’ to 5’ exonuclease proofreading domain, which can remove mis-paired nucleotides [33,34,35]. This domain safeguards the accuracy and integrity of the genome and its associated epigenetic modifications. However, the replicative DNA polymerases are susceptible to DNA damage, the distortions of the DNA template, and the secondary structures generated at the replication fork [36,37,38,39], leading to polymerase pausing and subsequently replication fork stalling and genome stress. Mutations or the functional deficiency of proteins that are involved in DNA replication and the resolution of stalled replication forks can also cause replication stress and genomic instability, and are associated with diseases [40]. For example, mutations of PRE-RC proteins are associated with the development of Meier Gorlin syndrome [41,42]. This may be because that the deficiency of PRE-RC proteins disrupts the assembly of the PRE-RC complex, thus inhibiting S-phase progression in cells [41]. On the other hand, deficiency of pol ε and GINS is associated with IMAGe (Intrauterine growth restriction, metaphyseal dysplasia, adrenal hypoplasia congenita, and genital anomalies) syndrome and immunodeficiency [43,44]. Also, Mutations in the helicases, including Bloom syndrome protein (BLM), Werner syndrome protein (WRN) and ATP-dependent DNA helicase QL4 (RecQL4) that mediate replication fork remodeling and restart can result in the development of Bloom, Werner and Rothmund-Thomson syndromes, respectively [45]. Bloom and Werner syndrome patients show aging-related symptoms including cancer predisposition, microcephaly, mental retardation, infertility, growth defects and premature aging, atherosclerosis, cataracts, osteoporosis and diabetes [46]. We have also included Table 1 with a list of the diseases that are associated with the deficiency of replication proteins

## 3. The Genome Stress Resulting from DNA Replication

There are varieties of sources that can cause genome stress during DNA replication, i.e., replication stress. These include physical impediments of replication fork progression induced by endogenous or/and exogenous DNA damaging agents [52], insufficient synthesis of histone proteins [53], and depletion of dNTPs [49,54,55]. In some occasions, DNA replication and repair enzymes can also create replication stress by inducing DNA lesions, such as abasic sites and ssDNA breaks, as well as by the incorporating damaged nucleotides through repair DNA polymerases [56,57,58,59]. 

Also, repeated DNA sequences in the genome that include microsatellites, minisatellites, isolated repeated motifs comprising homopolymers, elevation transposable elements, pseudogenes and terminal repeats, which constitute 50% of the human genome, can also cause genome stress during DNA replication. Among them, minisatellites and microsatellites are the major sources of causing “dynamic mutations,” i.e., repeat deletions and expansions in the genome [60]. These sequences can result in DNA replication fork stalling in the absence of exogenous genome stress [61,62]. They are susceptible to DNA damage and DNA strand breaks and thus known as DNA fragile sites that cause genomic instability [60,62]. Non-canonical or non-B form DNA structures are another source of causing genome stress through DNA replication stalling and DNA damage. The structures include triplex DNA, hairpins, DNA loops, Z-DNA, and G-quadruplexes [63,64,65,66]. They form the roadblocks of replicative and repair DNA polymerases to cause polymerase pausing impeding replication fork progression and DNA repair [63,64,65,66] 

### 3.1. The DNA Damage that Impedes the Fork Progression

DNA is under constant attack by a variety of endogenous and exogenous DNA damage agents, such as reactive oxygen species (ROS), UV among others, resulting in different types of DNA damage, including oxidized bases, modified sugars, abasic sites, DNA strand breaks, DNA-DNA and DNA-protein crosslinks and thymine dimers, which can result in replication fork stalling [52,56,57,67,68,69,70]. It is estimated that 10^4^ DNA base lesions are generated in the mammalian genome per day. These lesions can accumulate in the stalled replication fork while they are subject to DNA base excision repair (BER)/single-strand break repair (SSBR) [71,72]. However, repair of the lesions through BER/SSBR results in ssDNA breaks that can terminate the progression of polymerases at a replication fork. Also, unrepaired base lesions and abasic sites can directly block replication polymerases and helicases, leading to disassociation of polymerases from the template as well as helicase uncoupling, causing DNA strand breaks [57,71,73]. Bulky DNA damage, such as DNA-DNA and DNA-protein crosslinks and cyclobutane pyrimidine dimers (CPD) can also directly cause polymerase pausing and terminate replication fork progression [57,74,75,76,77,78]

### 3.2. Impediment of Replication Fork Progression by Gene Transcription 

DNA replication fork stalling can also be induced as a result of gene transcription. In the S phase, genes involved in DNA replication are highly expressed. This may result in a conflict between replication and transcription, i.e., transcription-replication conflicts (TRCs) when both replication and transcription occur simultaneously in the same DNA templates and collide head-on [64,79]. The collision slows down replication fork progression, leading to fork stalling and genome stress and genomic instability [64,79]. Furthermore, gene transcription can impede the replication fork progression through the formation of an R-loop that contains an RNA-DNA hybrid and a single-stranded non-template strand. The structure is involved in the disruption of genomic stability [64,80,81]. The RNA-DNA hybrid in an R-loop can be generated when nascent RNA transcripts reanneal to their template DNA, displacing the non-template strand into ssDNA, and this makes an R-loop become a potent barrier of co-transcription and replication [80,82]. R-loops can be stabilized by a deregulation of DNA replication and transcription proteins and factors [82,83,84]. The formation of R-loops is also facilitated by trinucleotide repeats including CAG, GAA, CGG repeats that can stabilize the DNA-RNA hybrid in the repeats [85,86,87,88]. The persistence of R loops in the GC-rich repeated sequences may facilitate somatic repeat expansion or deletion [89] by causing replication fork stalling, promoting the progression of trinucleotide repeat expansion diseases such as Huntington’s Disease (HD) and Friedreich’s Ataxia (FRDA) caused by CAG and GAA repeat expansions, respectively [85,86,89,90]. 

### 3.3. The Effects of dNTPs and Ribonucleotides on Replication Fork Progression

The progression of the replication fork and fidelity of DNA replication during S phase [49,54,55,91,92] is also regulated by the balance of dNTPs and the size of the nucleotide pool [55,93]. dNTPs are periodically synthesized and degraded at the different phases of the cell cycle [94,95,96]. A key step for the synthesis of dNTPs is the conversion of ribonucleotides triphosphate (NTPs) to deoxyribonucleotides (dNTPs) by ribonucleotide reductase (RNR), the rate-limiting enzyme for the synthesis of deoxynucleotide [94]. Inhibition of RNR by hydroxyurea (HU) depletes dNTPs, leading to replication fork stalling and genomic instability [55,93]. On the other hand, degradation/hydrolysis of dNTPs also regulates the balance dNTPs and nucleotide pool size to modulate the fidelity of replication and fork progression, impacting genomic stability. For example, knockdown of the dNTP triphosphohydrolase, sterile alpha motif and the HD-domain containing protein 1 (SAMHD1) in the G1 phase, disrupts the dNTP balance, stopping the progression of cell cycle and increasing cellular sensitivity to DNA damage [97,98]. Another important factor is the level of dUTP that can affect the fidelity of DNA replication. This is because replicative DNA polymerases cannot differentiate dUTP from dTTP [99,100]. Thus, the degradation of dUTP to dUMP by dUTP pyrophosphatase (dUTPase) plays a critical role in regulating dUTP to a low level in cells, ensuring the high fidelity of DNA replication. Thus, the rate of DNA replication fork progression and genomic stability is regulated by the balance of dNTPs and nucleotide pool size. Disruption of the balance between purine and pyrimidine can promote nucleotide misincorporations, which generate the source for replication fork stalling, DNA damage and genomic instability [101].

Interestingly, the incorporation of ribonucleotides by DNA polymerases is also associated with genomic instability. It is estimated that about 1 million ribonucleotides are incorporated into the genome during DNA replication by DNA polymerases [102]. Ribonucleotides are removed by RNase H2-mediated ribonucleotide excision repair (RER), which is the primary mechanism to remove ribonucleotides in the genome [103]. Accumulation of ribonucleotides resulting from the deficiency of RNase H2 can lead to replication stress and genomic stability [104]. Interestingly, under the deficiency of RNase H2, incorporated ribonucleotides are removed by DNA topoisomerase I and II [102,105]. However, the removal of incorporated ribonucleotides by topoisomerase can generate ssDNA and dsDNA breaks, deletion at repeated sequence, and genomic instability [106,107].

## 4. Cellular Responses to the Genome Stress from DNA Replication and Genome Instability

### 4.1. DNA Damage Response Signaling Induced by Stalled Replication Forks

To combat the unintended adverse consequences from stalled replication forks and the resulted DNA damage and maintain genomic instability and integrity, cells respond to the damage by initiating the DNA damage response signaling pathway that leads to cell cycle arrest [108]. The signaling pathway allows the coordination between DNA damage repair and replication fork processing for preventing stalled replication fork, DNA damage and strand breaks from being passed to the next phase in the cell cycle [57]. The DNA damage-response signaling pathway is activated through the activation of cell cycle checkpoints known as the DNA damage checkpoint (DDC) and DNA replication checkpoint (DRC). DDC is activated by DNA damage recognition, whereas DRC is activated by stalled replication forks [62,109,110,111]. For the cell cycle checkpoints, G1/S and G2/M [112], the G1/S phase checkpoint plays a major role in preventing the progression of cells carrying replication stress products, such as stalled fork and DNA damage [62,109,110]. Thus, the checkpoint allows DNA damage to be repaired in the S phase, so that DNA replication can proceed to the M phase. Both checkpoints demand that DNA damage generated during the G1 and G2 phases be repaired before the cell cycle can proceed to the next phase [2,51,113]. 

Activation of DRC is initiated by the slow progression of the replication fork along with the activation of the DNA replication checkpoints [114]. It has been shown that decreased replication fork progression by 5- to 10-fold leads to the activation of the ATR-mediated DNA damage response pathway. Further, it has also been found that a moderate level of replication stress induces the activation of ATR [115]. More severe replication stress induces the activation of both ATR and its downstream target pathways, such as FANC and CHK1 pathways [115,116,117]. Thus, cell response to replication stress through DRC is dependent on the ATR pathway [114,118,119]. Through the activation of the checkpoints, cell cycles are arrested, and DNA repair machineries are recruited to the damaged sites. Finally, DNA damage is repaired, and the stalled replication forks are resolved, allowing replication and cell division to proceed [120]. Thus, cell cycle checkpoints play vital roles in coordinating DNA damage repair and the resolution of stalled replication forks with cell cycle progression [121], leading to the maintenance of genome stability. 

### 4.2. Resolution of Stalled Replication Forks 

Stalled replication forks, if not resolved, will eventually result in replication forks collapse that can cause a series of severe consequences, such as DNA breakage and cell death. To avoid the scenario, stalled replication forks need to be resolved, and DNA replication needs to be restarted for cell survival. One strategy for eukaryotic cells, such as budding yeast to resolve stalled replication forks on the lagging strand, is to create new RNA primers at the downstream of DNA lesions that occur in the forks to restart DNA synthesis, a process named as repriming. It has been found that the repriming mechanism is used in the lagging strand DNA synthesis, as the synthesis of the Okazaki fragments is not affected by DNA damage and fork stalling as long as DNA is unwound continuously [122]. This is because the repriming process is initiated at the downstream of lesions [122]. In this process, a stalled DNA polymerase dissociates from the template strand and rebinds to the newly synthesized primer to synthesize DNA, thereby leading to the restart of stalled forks [123]. It has been found that discontinuous DNA synthesis can occur on both leading and lagging strands after UV damage in budding yeast, suggesting that the repriming mechanism is also used to resolve a stalled replication fork induced by DNA damage in the leading strand [123].

Also, eukaryotic cells can use a backup replication origin, i.e., the licensed replication origin to rescue stalled replication forks [124,125] because the reduced rate of replication fork progression can result in the accumulation of the ssDNAs, causing the uncoupling between DNA polymerase and helicase activities and large ssDNA gaps [126]. In this scenario, pol α-primase can be recruited to the ssDNA gaps and synthesize RNA primers to initiate DNA replication. 

**Figure 2:**
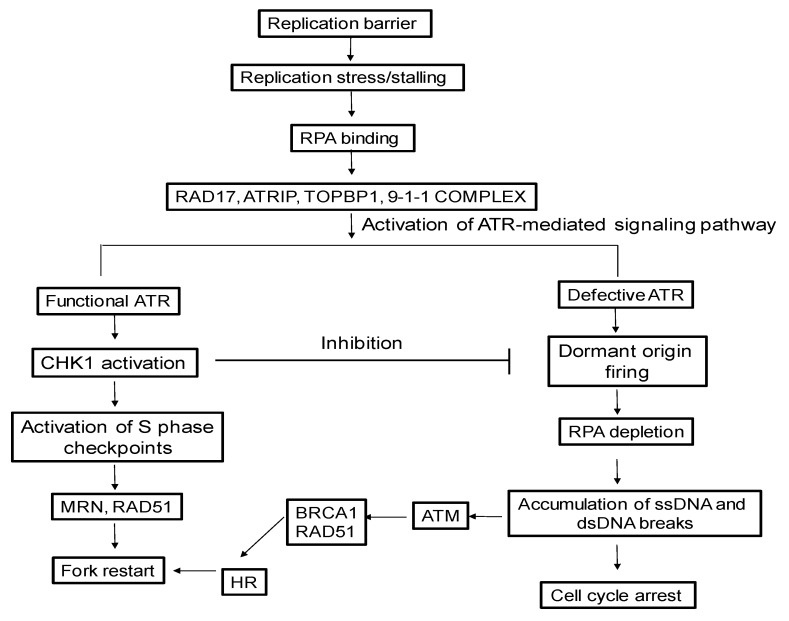
Ataxia telangiectasia and Rad3-related protein/(ataxia-telangiectasia mutated) serine/threonine kinase (ATR/ATM)-activated pathways for resolving stalled replication forks.

Since the recruitment of pol α-primase depends on TopBP1, which also involves in the activation of the ATR/MEC1 pathway [127], this suggests that the reactivation of the replication forks and the signaling pathway are coupled. The licensed origins of replication that are not activated during DNA replication are referred as the dormant origins of replication. They serve as a primary mechanism to restore replication when replication forks are stalled [128]. It has been estimated that about 20–30% of replication origins are activated during DNA replication [129]. Thus, the dormant origins bound by MCM helicases can serve as a backup for initiating the replication, thereby preventing replication stress, chromosome instability and tumorigenesis [130]. Dormant origin firing is regulated by ATR-mediated phosphorylation of FANCI [130]. In response to mild replication stress, unmodified FANCI triggers the firing of adjacent dormant origins to resolve stalled replication fork. In the case of severe replication fork stalling, dormant origin firing is inhibited by the phosphorylation of FANCI [130], and this provides more time for the stalled forks to be resolved, restarting DNA replication. Replication origin firing can also be modulated by claspin protein [131] that recruits Cdc7 kinase to the replication origin, which in turn phosphorylates MCM4, causing unscheduled origin firing in response to replication stress [129,130]. Inhibition of ATR can also result in an unscheduled origin firing, which can be modulated by Cdc7-mediated phosphorylation of MCM4 [129,130]. Figure 2 illustrated the Ataxia telangiectasia and Rad3-related protein/(ataxia-telangiectasia mutated) serine/threonine kinase (ATR/ATM)-activated pathways that are involved in resolution of stalled replication forks (Figure 2)

Stalled replication forks can also be broken down if not resolved, resulting in genomic instability and carcinogenesis [132,133]. To solve this issue, eukaryotes have evolved the MEC1/ATR pathway to combat the breakdown of the replication forks [134]. In addition, a stalled replication fork is protected by checkpoint and homologous recombination (HR) proteins [135]. Current models propose that the repair protein MRE11 expands the ssDNA gaps at a stalled replication fork behind the replisome, creating the substrate for the post-replicative repair. In contrast, RAD51 is loaded onto the stalled replication fork through BRCA2 to limit the expansion of the ssDNA gaps and protect the stalled forks from being broken [135].

### 4.3. Bypass of DNA Damage at Stalled Replication Forks 

When DNA damage occurs on stalled replication forks, they must be removed by DNA repair, or bypassed by DNA helicases and polymerases, allowing the restart, continuation, and completion of DNA replication. Failure to repair DNA lesions could result in DNA strand breaks, causing chromosomal rearrangement and cell death [136,137,138]. To ensure cell survival and the completion of the replication and cell cycle, cells may adopt lesion bypass if DNA damage at replication forks fails to be repaired [137]. The lesion bypass mechanisms include template switching, downstream repriming, recombination, lesion bypass through translesion synthesis (TLS) DNA polymerases and FANCJ [125,126,139]. However, the lesion bypass processes are usually error-prone, and can result in a rearrangement of chromosome and genomic instability associated with cancer. Here we discuss the lesion bypass of translesion DNA polymerases and the resulted genomic instability.

Unrepaired DNA lesions can be bypassed by TLS carried out by Y-family DNA polymerases [136,138,140,141] and some of the polymerases from the X- and A-family [142]. Since replicative DNA polymerases pause at DNA lesions, they are dislodged and substituted by TLS DNA polymerases, i.e., polymerase switching. This allows the incorporation of a nucleotide opposite the lesions by TLS polymerases for lesion bypass [40,109,138]. However, lesion bypass by TLS often results in nucleotide misincorporation and mismatches, causing mutations [138]. For example, incorporation of dAMP opposite 8-oxoG by TLS polymerases can induce the T→C transition mutation. DNA base lesions that can be bypassed by TLS polymerases are listed in Table 2. Also, TLS polymerases can incorporate damaged dNTPs and create mismatches to bypass a base lesion. It has been shown that oxidized dGTP can be incorporated opposite to dA on the template strand by TLS polymerases, inducing C→T transition and genomic instability [56,57,58]. It has been widely accepted that in the leading strand, DNA lesions need to be either repaired or bypassed by TLS polymerases for DNA synthesis to be continued during replication [140,143,144]. However, in the lagging strand, DNA lesions can be bypassed by TLS polymerases and repriming [123,136,138]. Thus, TLS polymerases play a crucial role in bypassing DNA lesions to maintain continuous DNA synthesis in the leading strand [140]. Upon the completion of DNA lesion bypass, TLS polymerases are dislodged by replicative polymerases through polymerase switching, restoring leading strand synthesis [145,146]. 

## 5. Cellular Responses to Genome Stress and Epigenetic Instability

### 5.1. Oxidative DNA Damage and Epigenetic Instability

Genome stress, including replication stress induced by oxidative stress and its resulted DNA damage, can also induce epigenetic instability. Typical epigenetic instability includes hypermethylation of the promoter of tumor suppressor genes (TSGs), and hypomethylation of non-promoter CpGs, such as repetitive elements and satellite DNA. 

The former causes transcriptional inactivation of TSGs, while the latter induces chromosomal instability and abnormal activation of oncogenes as well as mobile genetic elements. It has been found that a high level of ROS can lead to aberrant DNA hypermethylation in the gene promoter of TSGs, and their silencing suggesting an association between oxidative DNA damage with cancer-associated DNA methylation pattern changes. For example, exposure of hepatocellular carcinoma (HCC) cells to hydrogen peroxide leads to the hypermethylation of the promoter of the E-*Cadherin* gene via Snail-induced recruitment of histone deacetylase 1 (HDAC1) and DNA methyltransferase 1 (DNMT1) [152] to alter the DNA methylation pattern and chromatin structures. Further, oxidative DNA damage can inactivate TSGs through the recruitment of the polycomb repressive complex, which includes DNMT1, histone deacetylase (sirtuin-1) and histone methyltransferase to the CpGs containing 8-oxodGs [153]. It is possible that in responding to oxidative DNA damage, cells may use DNA hypermethylation to create heterochromatin in the genes, such as TSGs that are susceptible to DNA damage. This may shield DNA and protect them from further attack by DNA damaging agents. Interestingly, oxidative DNA damage can also result in DNA demethylation by inhibiting the binding of methyl-CpG binding protein 2 (MBP2) to methyl-CpGs, an epigenetic regulator that recruits DNMTs and histone HDAC to DNA [154]. This is because 8-oxodGs next to the 5mC at the CpGs inhibit the substrate binding of MBP [155,156]. Furthermore, the oxidized 5-methylcytosine, hydroxyl-5-methyl-cytosine can also decrease the binding affinity of MBPs resulting in DNA hypomethylation [157]. Thus, oxidative DNA damage can cause passive DNA demethylation, which in turn results in epigenetic instability leading to cancer and other diseases.

### 5.2. Histone Modifications at Stalled Replication Forks

**Figure 3:**
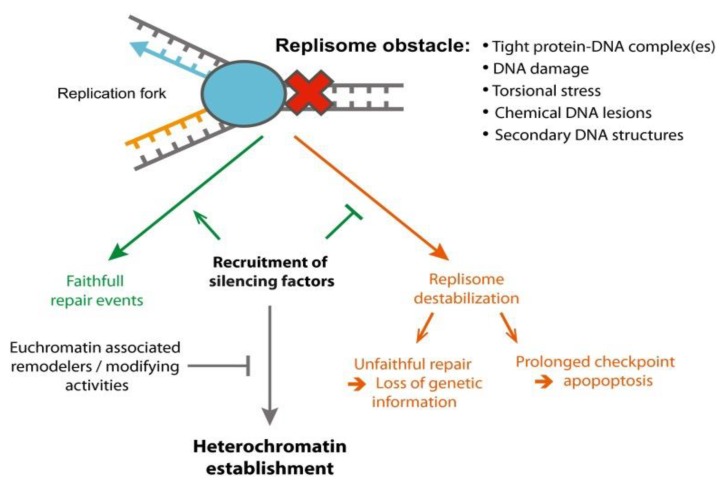
Heterochromatin formation during replication stress to prevent loss of genetic information [171].

Since double-helical DNA is wrapped around histone octamers that consist of H2A, H2B, H3 and H4 histone proteins, respectively [158], histone modifications that govern the structures of chromatin, i.e., opened (euchromatin) and closed (heterochromatin) conformation [159,160,161] play an important role in shielding DNA during cellular responses to DNA damage. It has been proposed that the formation of heterochromatin induced by genome stress such as replication fork stalling stops DNA replication (Figure 3) and prevents genomic instability. Histone tails are subject to different types of posttranslational modifications for the regulation of chromatin structures upon transcriptional activation or repression or chromatin opening or closing for DNA replication, and DNA damage and repair [162]. Specific histone modifications have also been identified as the response to replication stress [5]. The unscheduled firing of origin, fork stalling and repair of a collapsed fork can result in dramatic changes in chromatin structures. The methylation of newly synthesized histone proteins can be altered as a result of replication stress. This can alter the arrangement of old and newly-synthesized histone proteins, restoration of chromatin and patterning of epigenetic marks. It has been found that when a replication fork is stalled by genome stress, histones along with antisilencing factor 1 (Asf1) fail to be incorporated into chromatin, thereby increasing H3K9me1 [163]. Subsequently, methylation of H3K9 prevents histone acetylation, the active mark. H3K9me1 can also be further methylated into H3K9me3, the suppressive mark. These can then lead to the suppression of replication [5,163,164,165,166,167,168]. Further, histone methylation can recruit endonucleases to degrade the stalled replication fork. It has been found that methylation of H3K4 triggers MRE11-mediated degradation of replication fork, whereas H3K27me3 recruits MUS81 to cleave stalled forks [169,170]. The results indicate that cells adopt the epigenetic mechanisms to resolve stalled replication forks stalling.

Interestingly, the components of replisome, such as pol α, can bind to H2A and H2B [172]. Besides, MCM2 and pol ε can bind to H3 and H4 [173]. The interaction between the replication proteins and histones plays an important role in the redeposition of old histone to the newly-synthesized DNA. The redisposition of old histones helps the maintenance of the epigenetic marks of parental DNA in newly synthesized. Moreover, a challenge of DNA replication at non-B form DNA structures such as G quadruplexes can result in epigenetic instability [174]. Formation of the non-B form DNA structures such as G4 structures during replication can lead to an imbalance of the loading of old and new histones on the leading and lagging strands [175]. It has been shown that old histones are preferentially loaded on the lagging strand, whereas new histones are mainly loaded on the leading strand [175]. This results from the replication polymerases stalling at G4 structures in the leading strand. However, helicases can still keep unwinding the fork, allowing the continuation of the lagging strand synthesis. This subsequently results in the loss of H3K4me3, H3K9ac and H3k14ac marks in the regions at 4.5 kb downstream of the G4 structures in the leading strand [175,176]. The results indicate that cells adopt different epigenetic mechanisms to resolve stalled replication forks stalling.

### 5.3. DNA Damage and Modulation of miRNA Expression

DNA damage and its resulted replication stress can also alter the expression of microRNAs (miRNAs). MiRNA is short (18–22 nucleotide non-coding RNA molecules that base pair to the 3’ untranslated regions (UTR) of mRNAs) [177]. MiRNAs inhibit protein translation by promoting mRNA degradation or translation repression depending on the degree of their sequences complimentarily with those of their target mRNAs [177,178]. MiRNAs are involved in the regulation of cell proliferation, development, metabolism and gene expression [179,180]. Several classes of miRNAs are associated with the regulation of the genes of replication progression, cell cycle and DNA damage repair. Usually, miRNAs are deregulated by DNA damage [179,181,182,183]. It has been found that the miRNAs involved in cell cycle control can be upregulated by E2F [184]. MiRNAs that are deregulated by DNA damage include miR-34a, -34b and -34c. 

These mRNAs belong to the miR-34 family, and are upregulated in response to DNA damage. They are also the regulators of the expression of the checkpoint genes, such as E2F, CDK4, CDK6 and cyclin E2 [179,185]. In addition, miR-145a and miR-146b that target the tumor suppressor, BRCA1 are also upregulated upon double-strand DNA breaks [179,186]. MiR-155 and miR-21 that target mismatch repair proteins are upregulated during cellular responses to oxidative DNA damage induced by hydrogen peroxide and radiation [179,187]. On the other hand, miR-16 and mir-15 a/b that target the downregulators of checkpoint proteins, Cdc25a and Wip1, are also upregulated upon DNA damage. The Let-7 family miRNAs, *let-7i*, *mir*-*15b-16*-*2* and *mir-106b-25*, can also be induced by E2F. The miRNAs in this family are involved in limiting S phase entry as a result of genome stress, thereby preventing mutagenesis [184]. Also, miRNAs can downregulate MCM2-7 in a *Trp53*-dependent manner [183]. The roles of miRNA in mediating cellular response to genome stress warrant further studies in the future.

## 6. Conclusions

DNA damage and genome stress, including DNA replication stress, can cause genomic and epigenomic instability, which are associated with many diseases such as cancer. The studies summarized here have pointed to a direct link among DNA damage, genome stress, such as replication stress and genomic and epigenomic instability. Since genome stress triggers the alteration of genetic and epigenetic information that can be passed to the next generation, it is important to further explore how genome stress, such as replication stress, can crosstalk with DNA methylation, chromatin structures and miRNA expression in the context of a variety of diseases. 

## Figures and Tables

**Table 1:** Proteins involved in DNA replication, repair, and replication stress response and associated diseases.

DNA Repair Protein	Function	Human Diseases
CDT1	Facilitates MCM loading on origins	Meier-Gorlin syndrome [40]
Pre-RC (CDT1, ORC1-ORC6, Cdc6, MCM2-7)	Recruitment of DNA polymerase and phosphorylation by both the Cdc7/Dbf4 and CDK2-cyclin A protein kinases	Meier-Gorlin syndrome [40]
Nbs1	ATR/ATM activation	Nijmegen breakage syndrome [40]
Rad50	ATR/ATM activation	Nijmegen breakage syndrome-like disorder [40]
RecQL4	DNA remodeling, replication fork structure resolution	Rothmund-Thomson syndrome [40,47]
RNase H2	Removal of embedded ribonucleotides Resolution of RNA-DNA hybrid	Aicardi-Goutières syndrome [48]
Senataxin	Resolution of RNA-DNA hybrid	Amyotrophic lateral sclerosis [40]
Mre 11	ATM/ATR activation	Ataxia-telangiectasia-like diseases [40]
BLM	DNA remodeling, replication fork stall resolution	Bloom syndrome [49]
FANC family	DNA inter-strand cross-link repair	Fanconi anemia [40,50]
FANCD2	Replication fork protection	Fanconi anemia [40,50]
WRN	DNA remodeling, replication fork structure resolution	Werner syndrome [40]
BRCA1, BRCA2	Checkpoint mediators, DNA repair and recombination	Breast and ovarian carcinoma [51]
MSH2 and MLH1	DNA mismatch repair	Colorectal cancer [51]

**Table 2:** Translesion DNA polymerases and their bypass of DNA base lesions.

Proteins	DNA Lesions	Nucleotide Preference of Lesion Bypass
Pol η	Thymine dimer	Prefer dA, followed by dG >dT>dC [147]
	8-oxoG	Prefer dC and dA [148]
	Acetyl amino fluorene-dG	Prefer dC followed by dG > dT> dA [147]
	N^6^-ethenodeoxyadinosine	Prefer dT followed by dA >dG>dC [149]
	Abasic-site	Prefer A [147]
Pol қ	Thymine dimers	Could not bypass [150]
	N^6^-ethenodeoxyadinosine	Prefer dT followed by dA >dC>dG [149]
	Abasic site	Prefer dA followed by dG >dT>dC [150]
Pol ι	Thymine dimer	Prefer T and A followed by dG >dC [151]
	Abasic site	Prefer dA [151]

## Abbreviations

ASF	Alternative splicing factor 1
ATM	(ataxia-telangiectasia mutated) serine/threonine kinase
ATR	Ataxia telangiectasia and Rad3-related protein
ATPIP	ATR interacting protein
BRCA1	Breast cancer 1
BLM	Bloom syndrome
BRCA2	Breast cancer type 2 susceptibility protein
Cdc45	Cell division cycle 45
Cdc6	Cell Division Cycle 6
CDK	Cyclin dependent kinase
Cdt1	Cdc10-dependent transcript 1
Chk1	Checkpoint kinase 1
CMG	CDC45-MCM-GINS
CPDs	Pyrimidine dimers
Ctf4	Chromosome transmission fidelity 4
dAMP	Deoxyadenosine monophosphate
Dbf4	Dumbbell former 4
DDC	DNA damage checkpoint
DDK	Dbf4 dependent kinase
DDR	DNA damage response
DDSBs	DNA double-strand breaks
DGCR8	DiGeorge syndrome critical region 8)
dNDPs	Deoxyribonucleotides diphosphate
DNMT1	DNA methyltransferase 1
dNTPs	Deoxynucleotide triphosphates
DRC	DNA replication checkpoint
dUMP	Deoxyuracilmonophosphate
dUTP	Deoxyuraciltriphosphate
dUTPase	dUTP pyrophosphatase
FANCJ	Fanconi Anemia complementation group J Protein
FEN 1	Flap endonuclease 1
FRDA	Friedreich’s ataxia
FXN	Frataxin gene
GINS	Go-ichi-ni-san
HCC	Hepatocellular carcinoma
HD	Huntington’s disease
HDAC1	Histone deacetylase 1
HR	Homologous recombination
HU	Hydroxyurea
IMAGe	Intrauterine growth restriction, metaphyseal dysplasia, adrenal hypoplasia, and genital anomalies
ICLs	Interstrand DNA crosslinks
MCM	Minichromosome maintenance protein
MEC1	Mitosis entry checkpoint 1
MRE11	Microhomology-mediated end-joining 11
ORC	Origin recognition complex
PCNA	Proliferating cell nuclear antigen
PreRC	Pre-replication complex
PTMs	Post-transcriptional modifications
RecQL4	RecQ like helicase 4
RFC	Replication factor C
RISC	RNA-induced silencing complex
RNR	Ribonucleotide reductase
ROS	Reactive oxygen species
SF2	Splicing factor 2
SRSF1	Serine/arginine-rich splicing factor 1
ssDNA	Single strand DNA
TLS	Translesion polymerases
TopBP1	Topoisomerase II Binding Protein 1
TRCs	Transcription-replication conflicts
TSGs	Tumor suppressor genes
UTR	Untranslated region
SAMHD1	SAM domain and HD domain-containing protein 1
WRN	Werner syndrome
